# The Effect of Different pH and Temperature Values on Ca^2+^, F^−^, PO_4_^3−^, OH^−^, Si, and Sr^2+^ Release from Different Bioactive Restorative Dental Materials: An In Vitro Study

**DOI:** 10.3390/polym17050640

**Published:** 2025-02-27

**Authors:** Angelo Aliberti, Fabiana Di Duca, Maria Triassi, Paolo Montuori, Stefano Scippa, Mirko Piscopo, Pietro Ausiello

**Affiliations:** 1 Department of Neurosciences, Reproductive and Odontostomatological Sciences, University of Naples Federico II, Via Sergio Pansini 5, 80131 Naples, Italy; ange.aliberti@studenti.unina.it (A.A.); mirko.piscopo.98@gmail.com (M.P.); 2 Department of Public Health, University of Naples Federico II, Via Sergio Pansini 5, 80131 Naples, Italy; fabianadiduca91@gmail.com (F.D.D.); maria.triassi@unina.it (M.T.); paolo.montuori@unina.it (P.M.); stefanoscippa923@gmail.com (S.S.); 3 Interdepartmental Research Centre in Health Management and Innovation in Healthcare (CIRMIS), Via Sergio Pansini 5, 80131 Naples, Italy

**Keywords:** dental materials, ions, glass ionomer cements, calcium, fluoride, strontium, phosphate

## Abstract

Bioactive restorative materials are crucial for promoting remineralization and protecting dental tissues through ion release. This study examines how pH and temperature influence the short- and long-term ion (F^−^, Ca^2+^, Sr^2+^, OH^−^, Si, and PO_4_^3−^) release from seven commercial materials: *Cention Forte Filling Material*, *Cention Primer*, *Stela Self Cure*, *Riva Light Cure HV*, *Riva Self Cure*, *Equia Forte HT Fil*, and *Fuji IX GP Fast*. Disks were prepared according to the manufacturers’ instructions; immersed in buffer solutions at pH 4.8, 6.8, and 8.8; and stored at 37 °C and 44 °C. Ion release was measured after 1, 7, and 28 days using ion chromatography and mass spectrometry. Results revealed that ion release was significantly affected by pH, temperature, and exposure time. The highest fluoride (40.14 ± 0.32 mg/L) and calcium (74.23 ± 0.37 mg/L) releases were observed in *Riva Light Cure* at pH 4.8 and 44 °C after 28 days, with the highest strontium release (5.87 ± 0.06 mg/L) occurring under the same conditions. In contrast, silicon release peaked in *Cention Forte Filling* (31.72 ± 0.68 mg/L) at pH 4.8 and 37 °C. These findings highlight the impact of environmental factors on material performance, assisting clinicians in selecting optimal restorative materials for long-term dental health.

## 1. Introduction

Dental restorative procedures are based on the use of different techniques, classified as direct and indirect, and materials, depending on the specific clinical needs.

The main clinical objectives are to preventively avoid dental caries development and lastly to restore reduced masticatory function. The most used materials for direct methods are as follows: ions releasing materials (F^−^, Ca^2+^, and Sr^+^) by primary prevention protocols and resin-based composites, which are used through adhesive techniques and performed in both incremental layering and bulk-fill modes [1].

However, in recent years, some of the research on new dental materials has focused on bioactive materials that not only restore masticatory function but also interact with the hard tissues of teeth and play an important role in preventing dental secondary caries and in maintaining long-term dental health. This is because these materials, such as glass ionomer cements and alkasites, are designed to promote the remineralization of dental tissues and inhibit the formation of caries through active biological mechanisms [2].

Recent studies have shown that alkasites release significant amounts of fluoride (F^−^) and calcium (Ca^2+^) ions. This release contributes to restoring the integrity of the tooth enamel and balancing the pH of the oral environment [3]. In addition, this mechanism also ensures stable preservation of the microenvironment around the restoration remains stable and protective and elevates the material’s ability to resist demineralization and contributing to long-term oral health [4]. Other studies have shown that glass ionomer cements also support the remineralization of demineralized dentin by releasing ions through the biomineralization process. Recent results have shown the formation of carbonate apatite with an increase in the Ca/P ratio [5]. This process occurs because glass ionomer cements have a chemical composition that acts as a buffer against the pH of the surrounding fluid. Indeed, when exposed to an acidic environment, as in caries, these materials neutralize the acidity and stabilize the pH to a weak acid. This alkalization may be related to the release of various ions by these materials, which, due to their chemical properties, can reduce the acidity of the environment [6].

Demineralization, which plays a fundamental role in the development of primary and secondary caries, can be prevented by increasing the pH through the release of high concentrations of ions in acidic environments. This process reduces the amount of free hydrogen ions and helps to neutralize acidity [7]. Therefore, the scientific literature has shown that bioactive materials can induce the formation of apatite and help stabilize the pH in conditions where the oral cavity becomes acidic, such as in caries development. This property, therefore, strengthens the resistance of the enamel to demineralization processes [8].

For these reasons, the release of ions such as calcium (Ca^2+^), fluoride (F^−^), hydroxide (OH^−^), strontium (Sr^2+^), and phosphate (PO_4_^3−^) by bioactive substances is crucial in the prevention of dental caries. All this information has been studied in pediatric dentistry supporting the use of these substances through clinical protocols with toothpastes and mouthwashes.

Fluoride is particularly advantageous because its long-term sustained release has a significant effect on secondary caries formation. Glass ionomer cements have the property of prolonged fluoride release by ion exchange mechanism [9]. Indeed, the release of fluoride from such bioactive materials contributes to increased resistance to acids by promoting the formation of fluorapatite on the tooth surface, which is less soluble than hydroxyapatite [10].

There are studies showing that even at very low concentrations, it is effective in inhibiting enamel demineralization and promoting remineralization, especially in cycles of pH variation, where the presence of fluoride is essential and contributes to slowing the progression of caries [11]. In this context, it has been shown that fluoride-containing bioactive glasses can have excellent synergistic interaction with calcium and phosphate ions. This synergistic effect is important because it contributes to the formation of a protective layer under acidic conditions, thereby further increasing biological activity [12].

Strontium is an element that has been shown to be useful in the prevention and treatment of dentine hypersensitivity and contributes to caries control. This alkaline earth metal has similar properties to calcium. Therefore, it can be used as a substitute for enamel formation [13]. Indeed, the conversion of hydroxyapatite to strontium apatite has demonstrated its ability to increase the resistance of teeth to acid [14].

Furthermore, the substitution of strontium in remineralizing resin-based composites not only helps in the treatment of dentine hypersensitivity, but may also confer antibacterial properties, thus reducing the risk of caries through the prolonged release of ions [15]. In fact, scientific studies have shown that these cations reduce acid production via *Streptococcus mutans* in bio membranes by increasing the pH of the surrounding environment [16].

The release of cations such as calcium by bioactive substances is of great importance as it helps to reduce material loss, decrease demineralization events, and prolong the remineralization time [17]. In addition, since calcium ions act as stabilizing agents within the demineralized collagen matrix, they are an essential component of maintaining mineral homeostasis in dental tissue [18]. Very small amounts of calcium are required to create a saturated environment conducive to mineral precipitation [19]. In fact, calcium is also added to resin-based composite resins due to its ability to release minerals that remineralize the tooth structure [20]. Minimal changes in calcium concentration have a greater effect than similar changes in phosphate concentration. Furthermore, in vitro experiments have shown that calcium is approximately 20 times more effective than phosphate in inhibiting enamel dissolution, suggesting that calcium ion concentration is a limiting factor in enamel remineralization [21].

The release of hydroxyl ions is also very beneficial, contributing significantly to lowering the pH and acting as an alkalizing agent [22]. Scientific studies have also shown that a decrease in pH increases the likelihood of fracture of dental tissue and reduces mechanical properties [23]. The acidic environment exacerbates microleakage and can lead to an increased presence of cariogenic bacteria, compromising the structural integrity of the dental material and surrounding dental tissue [24].

Furthermore, these ions have antimicrobial effects through inhibition of bacterial enzyme systems [25]. Studies have shown that hydroxyl ions can alter the integrity of bacterial cytoplasmic membranes, disrupting both lipid structure and nutrient transport. These ions break down lipopolysaccharides in the cell walls of Gram-negative bacteria, contributing to the reduction of toxins and inflammation [26].

Phosphate ions play an important role in dentin remineralization, binding to calcium and promoting apatite formation. To facilitate this process, it is fundamentally important that the materials used for restoration release mineral ions such as calcium and phosphate and act as a stable site for the nucleation process [27]. The remineralization and protective effect caused by this ion release of bioactive materials is not limited to the tissue immediately after placement of the restoration, but extends to other areas of the oral cavity, such as the floor of deep cavities with marginal leakage, the margin of the restoration and non-carious class V lesions where the cementum is exposed. Various studies have shown that [28]. Additionally, bioactive materials that release phosphate ions can cooperate in maintaining the structural stability of the tooth and in protecting the restoration from marginal lesions, offering a long-term preventive effect against secondary caries [29].

The objective of this study is to evaluate in laboratory conditions the short- and long-term release of fluoride (F^−^), calcium (Ca^2+^), hydroxyl (OH^−^), strontium (Sr^2+^), silicon (Si), and phosphate (PO_4_^3−^) ions from different restorative materials at different pH levels and temperatures. It aims to fill an important gap in the existing literature by focusing on different terms in vitro simulations that more accurately replicate the conditions encountered by restorative materials in clinical settings.

The study aims to determine how some environmental factors, such as pH and temperature, influence the ion release properties of these materials, and how these factors impact their bioactive properties.

## 2. Materials and Methods

### 2.1. Sample Type

Seven types of restorative materials available on the market were analyzed. Details regarding the selected materials and their compositions are outlined in Table 1. The preparation of dental material specimens followed the guidelines provided by the manufacturers. Specimens were prepared according to the manufacturers’ instructions: *Cention Forte Filling Material* (Ivoclar), *Equia Forte HT Fil* (GC Europe), *Stela Self Cure* (SDI), *Riva Self Cure* (SDI) and *Fuji IX GP Fast* (GC) were prepared using self-curing methods. *Riva Light Cure HV* (SDI), and *Cention Primer* (Ivoclar) were prepared using light curing. All materials were prepared using a stainless-steel cylinder with a 10 mm diameter and a 2 mm thickness. Each specimen was mixed by a 3MTM ESPETM CapMixTM mixer (3M ESPE, Seefeld, Germany) for 10 s and immediately applied to the cylinders. The samples were gently compressed with a celluloid strip and a smooth condenser to prevent the formation of air bubbles and to obtain a smooth and flat surface. No coating varnish was applied at the top. After 5 min, they were removed, and the surface was polished with 800 grit abrasive. paper using a water-cooled rotating polishing machine (Ecomet 30, Buehler Ltd., Lake Bluff, IL, USA).

### 2.2. Testing Conditions of the Materials

The analyses were conducted following the methodology described in a previous study [1]. In summary, samples (n = 3 for each material) from each material were im-mersed in 50 mL of buffer solution at three distinct pH levels (4.8, 6.8, and 8.8) and in-cubated in temperature-controlled laboratory ovens (Precision Thelco, Thermo Fisher Scientific, Waltham, MA, USA) set at 37 °C and 44 °C. A 1M CH_3_COOH/CH_3_COONa_3_ × H_2_O buffer was prepared for the acidic environment at pH 4.8, a Phosphate-Citrate buffer for the neutral environment at pH 6.8, and a 1M Tris-HCl buffer was used for the basic environment at pH 8.8. The materials were left in the respective soaking solutions for durations of 24 h, 7 days, and 28 days before being transferred to 50 mL Falcon tubes for subsequent analysis.

#### 2.2.1. pH Measurements

For pH measurements, 5 mL of soaking solution was collected from each sample and placed in 15 mL Falcon. A digital pH meter (Mettler Toledo-SevenExcellence pH/Cond meter S470-Std-K), calibrated with standard solutions (at pH = 4.0, pH = 7.0, pH = 9.0), was used.

#### 2.2.2. Fluoride and Phosphate Ion Release

The release of cumulative fluoride (F^−^) and phosphate (PO_4_^3−^) ions was assessed by ion chromatography. Specifically, 1 mL of soaking solution from each material sample (n = 3 per material) was placed in a 1.5 mL vial for analysis. The ion concentration was determined using a DIONEX Integrion HPIC^TM^ ICS1100 ion chromatography system (Thermofisher, Bremen, Germany), supplied with an IonPac AS27 RFIC^TM^ (4 × 250 mm) (Thermofisher) analytical column. Then, 25 µL of each sample was injected at 1.0 mL/min, and ion concentrations were determined from the retention times of the chromatographic peaks, using calibration curves prepared with standard solutions. To be more specific, for F^−^, the calibration curves were obtained using six calibration points at concentrations of 1.0, 2.5, 5.0, 10.0, 25.0, and 50.0 mg/L. For PO_4_^3−^, the calibration range was 0.5–50 mg/L. The limits of quantification (LoQs) were 1.0 and 0.5 mg/L, respectively.

#### 2.2.3. Assessment of Calcium, Silicon, and Strontium Concentrations

For the evaluation of calcium (Ca^2+^), silicon (Si), and strontium (Sr), samples of each material (n = 3) (soaked in 10 mL of solution) were acidified with 1% HNO_3_/HCl (3:1% *v*/*v*) and analyzed by mass spectrometry with inductively coupled plasma (ICP-MS) using a trace elemental analyzer (Thermo Scientific^TM^ ICAP^TM^ RQ) and an inductively coupled plasma mass spectrometer (Q-ICP-MS), operated by software Qtegra^TM^ Intelligent Scientific Data Solution^TM^ (version 2.10.3324.131). The operating conditions of the equipment (Q-ICP-MS) were optimized using a tuning solution (Ba, Bi, Ce, Co, In, Li, and U 1.00 µg/L, purchased from Thermo Scientific). The analyses were conducted in KED (kinetic energy discrimination) mode, utilizing helium as the collision gas. The concentrations were determined using a calibration curve (CertiPUR^®^, Merck, Darmstadt, Germany) with a satisfactory correlation coefficient (r^2^) greater than 0.98. In detail, for Ca^2+^ and Si, the calibration range was 1–50 mg/L; for Sr, it was 0.02–10 mg/L. Consequently, the limits of quantification (LoQs) were 1.0 mg/L for Ca and Si, and 0.02 mg/L for Sr, respectively.

### 2.3. Statistical Analysis

For the statistical analysis, STATA 14.0 software (College Station, TX, USA) was used. Descriptive statistics were conducted to determine the mean concentrations, standard error, and the minimum and maximum values for each material. The Shapiro–Wilk test was used to assess the normality of variable distribution. Mixed-effects models (MEMs) were applied to compare differences between groups based on pH, temperature, and exposure time. The statistical significance of each independent variable was assessed using the Wald test, with differences considered significant at *p* < 0.05.

## 3. Results

### 3.1. pH Measurements Results

**Figure 1:**
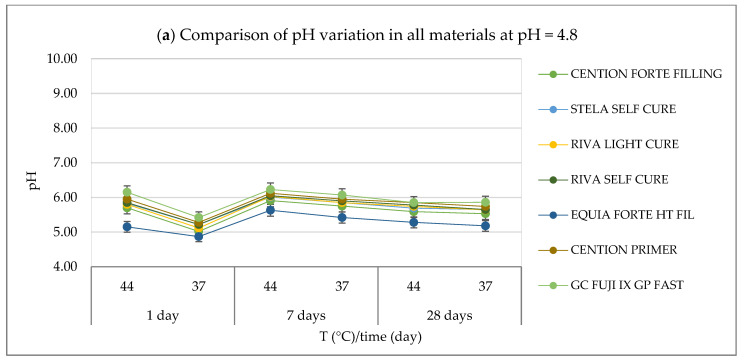

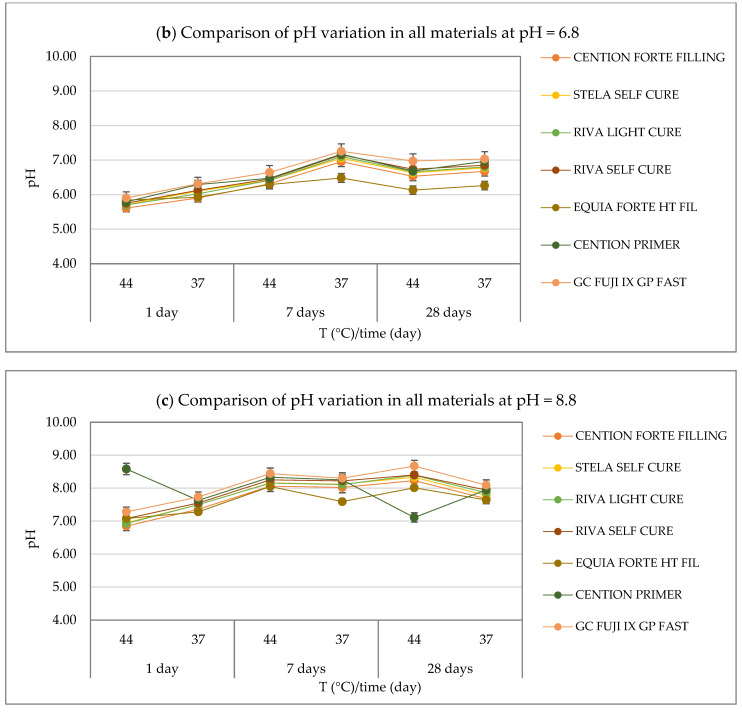
Comparison of pH variation in all materials at (**a**) pH = 4.8, two temperatures (44 and 37 °C), and at three different observation times (24 h, 7 days, and 28 days); (**b**) pH = 6.8, two temperatures (44 and 37 °C), and at three different observation times (24 h, 7 days, and 28 days); (**c**) pH = 8.8, two temperatures (44 and 37 °C), and at three different observation times (24 h, 7 days, and 28 days).

The results of the pH measurements carried out for the various materials under the established conditions (acidic, basic, and neutral pH; two temperatures: 37 °C and 44 °C; and three different observation times: 24 h, 7 days, and 28 days) are graphically illustrated in Figure 1 and detailed in Table S1.

For all materials, a similar behavior was observed, with relatively small variations in pH values. Under acidic conditions (pH = 4.8), all materials showed an increase of approximately one pH unit compared to the initial value (Figure 1a). Specifically, for the *Cention Forte Filling Material*, the pH values ranged from 5.02 ± 0.06 at 37 °C after 24 h to 5.91 ± 0.07 at 44 °C after 7 days, with an average of 5.58 ± 0.31. Similarly, for *Stela Self Cure*, the observed pH values varied between 5.22 ± 0.03 at 37 °C after 24 h and 6.01 ± 0.07 at 44 °C after 7 days, with a mean value of 5.71 ± 0.27. *Riva Light Cure HV* showed a range of 5.12 ± 0.04 to 6.03 ± 0.03 under the same conditions, with an average of 5.68 ± 0.31. In the case of *Riva Self Cure*, the pH values were between 5.22 ± 0.07 and 6.05 ± 0.08, observed at 37 °C after 24 h and at 44 °C after 7 days, respectively, yielding a mean of 5.74 ± 0.29. The *Equia Forte HT Fil* material exhibited slightly lower values, ranging from 4.87 ± 0.03 at 37 °C after 24 h to 5.63 ± 0.04 at 44 °C after 7 days, with an average of 5.25 ± 0.26. *Cention Primer* material showed higher values, with pH measurements ranging from 5.28 ± 0.07 to 6.12 ± 0.06 under the same conditions, corresponding to an average of 5.82 ± 0.29. Finally, *GC Fuji IX GP Fast* material presented the highest observed pH values, ranging between 5.42 ± 0.07 and 6.23 ± 0.04, with an average of 5.93 ± 0.29.

Even in a neutral environment (pH = 6.8), only small pH variations were recorded (Figure 1b). However, unlike in the acidic environment, all materials initially showed a decrease in pH of approximately one unit during the first 24 h, followed by a stable behavior thereafter. Specifically, for the *Cention Forte Filling Material*, pH values ranged from 5.61 ± 0.08 at 44 °C after 24 h to 6.95 ± 0.08 at 37 °C after 7 days, with an average value of 6.33 ± 0.50. Similarly, for *Stela Self Cure*, the pH ranged from 5.71 ± 0.06 at 44 °C after 24 h to 7.05 ± 0.04 at 37 °C after 7 days, resulting in an average value of 6.45 ± 0.48. The material *Riva Light Cure HV* exhibited a similar trend, with pH values ranging from 5.70 ± 0.07 at 44 °C after 24 h to 7.10 ± 0.06 at 37 °C after 7 days, and an average value of 6.45 ± 0.52. For *Riva Self Cure*, pH values were observed between 5.75 ± 0.07 at 44 °C after 24 h and 7.15 ± 0.08 at 37 °C after 7 days, giving an average value of 6.51 ± 0.51. In the case of *Equia Forte HT* Fil, pH values ranged from 5.84 ± 0.04 at 44 °C after 24 h to 6.48 ± 0.04 at 37 °C after 7 days, with a mean of 6.15 ± 0.24. For *Cention Primer*, the pH ranged from 5.79 ± 0.03 at 44 °C after 24 h to 7.17 ± 0.08 at 37 °C after 7 days, with an average value of 6.56 ± 0.49. Finally, the *GC Fuji IX GP Fast* material showed pH values ranging between 5.90 ± 0.07 at 44 °C after 24 h and 7.25 ± 0.04 at 37 °C after 7 days, with an average of 6.68 ± 0.51.

In a basic environment (pH = 8.8), a behavior like that observed in the acidic environment was noted, with an initial decrease in pH of approximately two units during the first 24 h, followed by a subsequent increase, returning to the starting value. Specifically, for the *Cention Forte Filling Material*, pH values ranged from 6.85 ± 0.04 at 44 °C after 24 h to 8.23 ± 0.04 at 44 °C after 28 days, with an average value of 7.70 ± 0.52. For *Stela Self Cure*, pH values ranged from 6.95 ± 0.06 at 44 °C after 24 h to 8.33 ± 0.04 at 44 °C after 28 days, with a mean value of 7.81 ± 0.52. Similarly, for *Riva Light Cure*, pH values ranged from 6.93 ± 0.08 at 44 °C after 24 h to 8.39 ± 0.07 at 44 °C after 28 days, with an average value of 7.82 ± 0.53. For *Riva Self Cure*, pH values ranged between 7.08 ± 0.06 at 44 °C after 24 h and 8.40 ± 0.05 at 44 °C after 28 days, with a mean of 7.91 ± 0.50. In the case of *Equia Forte HT Fil*, pH values varied from 7.08 ± 0.05 at 44 °C after 24 h to 8.05 ± 0.07 at 44 °C after 7 days, with an average value of 7.61 ± 0.39. For *GC Fuji IX GP Fast*, pH ranged from 7.28 ± 0.04 at 44°C after 24 h to 8.67 ± 0.05 at 44 °C after 28 days, with a mean of 8.08 ± 0.51. Only for *Cention Primer* were decreasing pH values observed as the observation time increased. Specifically, the pH values ranged from 7.11 ± 0.07 at 44 °C after 28 days to 8.58 ± 0.04 at 44 °C after 24 h, with an average value of 7.96 ± 0.54.

In conclusion, despite variations across different materials and conditions, the pH changes observed were relatively consistent, with all materials exhibiting similar trends. Under acidic conditions, there was an approximate increase of one pH unit, while in neutral and basic environments, an initial decrease in pH was followed by stabilization or a return to baseline values. These patterns suggest that the tested materials demonstrate stable pH profiles over time across various environmental conditions, with some varia-tions indicating differing susceptibilities to pH changes. These findings provide valuable insight into the long-term behavior of these materials, which is essential for under-standing their potential applications in diverse fields.

### 3.2. Release of Fluoride and Phosphate Ions

**Figure 2:**
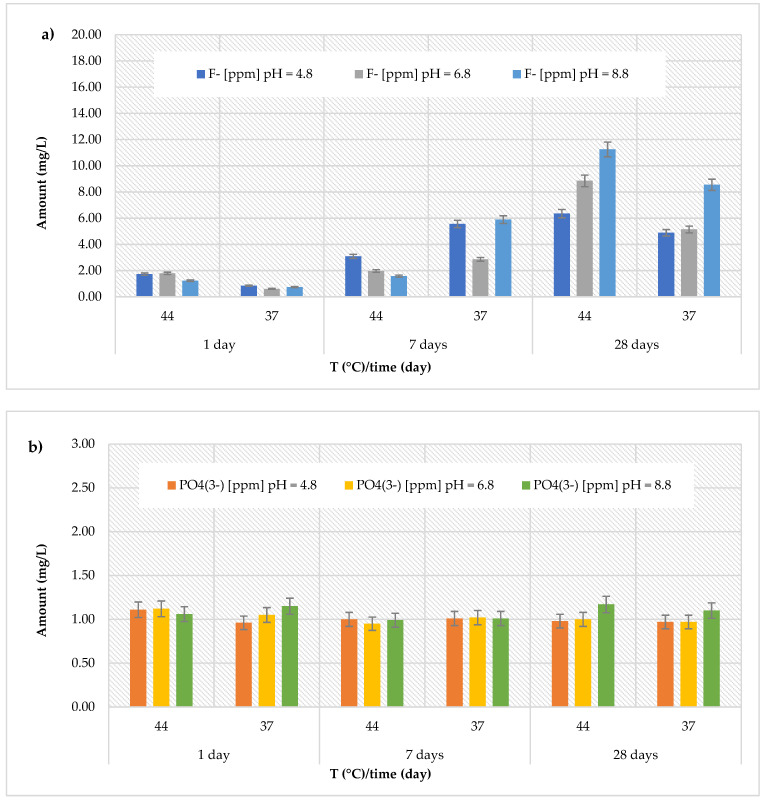
(**a**) Comparison of [F]^−^ release from *Cention Forte Filling Material* across three different pH levels, two temperatures (44 and 37 °C), and at three different observation times (24 h, 7 days, and 28 days); (**b**) comparison of [PO_4_^3−^] release from *Cention Forte Filling Material* across three different pH levels, two temperatures (44 and 37 °C), and at three different observation times (24 h, 7 days, and 28 days).

**Figure 3:**
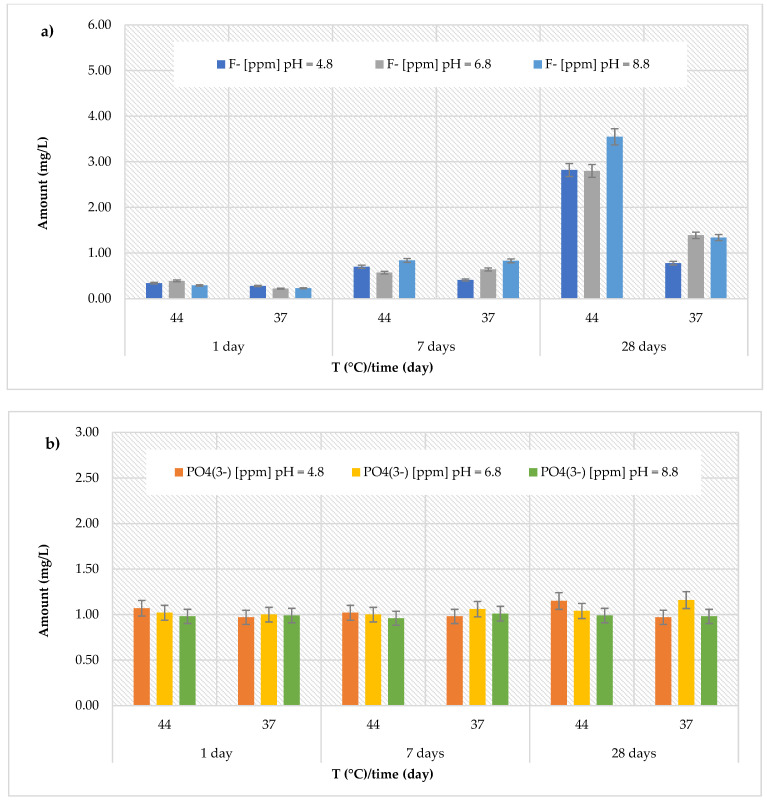
(**a**) Comparison of [F]^−^ release from *Stela Self Cure* material across three different pH levels, two temperatures (44 and 37 °C), and at three different observation times (24 h, 7 days, and 28 days); (**b**) comparison of [PO_4_^3−^] release from *Stela Self Cure* material across three different pH levels, two temperatures (44 and 37 °C), and at three different observation times (24 h, 7 days, and 28 days).

**Figure 4:**
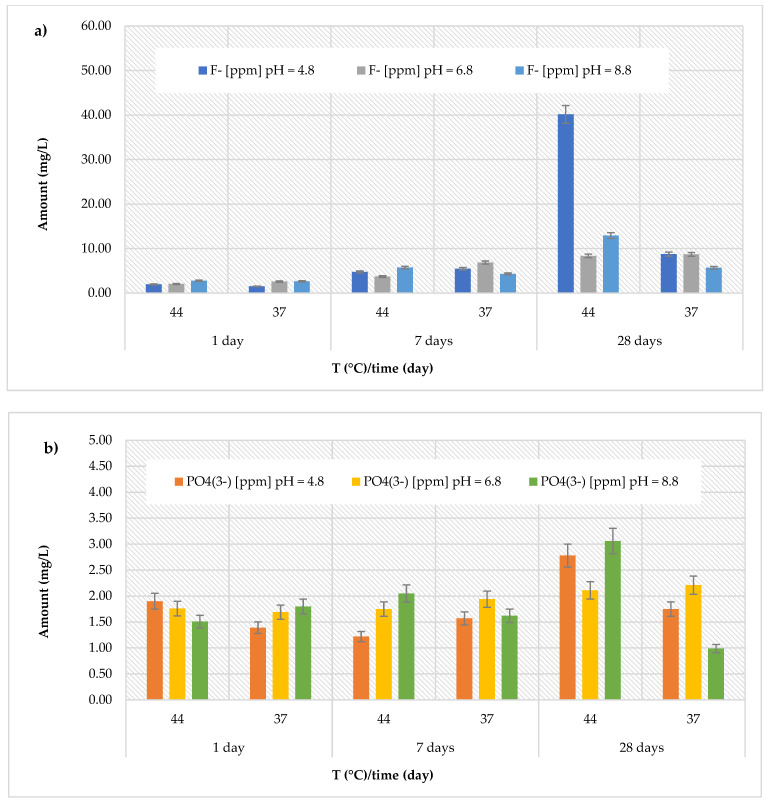
(**a**) Comparison of [F]^−^ release from *Riva Light Cure HV* material across three different pH levels, two temperatures (44 and 37 °C), and at three different observation times (24 h, 7 days, and 28 days); (**b**) comparison of [PO_4_^3−^] release from *Riva Light Cure HV* material across three different pH levels, two temperatures (44 and 37 °C), and at three different observation times (24 h, 7 days, and 28 days).

**Figure 5:**
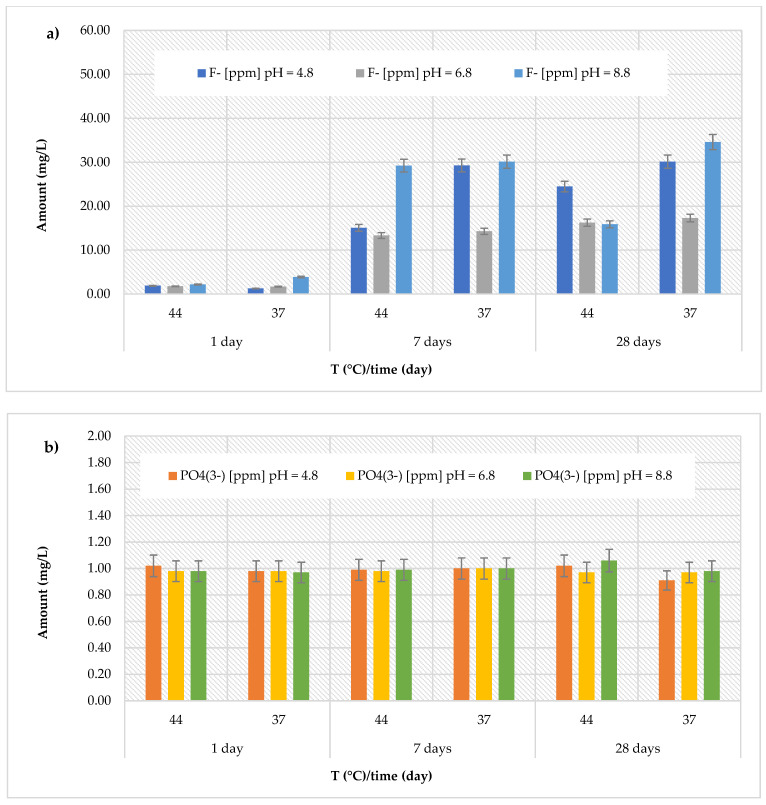
(**a**) Comparison of [F]^−^ release from *Equia Forte HT Fil* material across three different pH levels, two temperatures (44 and 37 °C), and at three different observation times (24 h, 7 days, and 28 days); (**b**) comparison of [PO_4_^3−^] release from *Equia Forte HT Fil* material across three different pH levels, two temperatures (44 and 37 °C), and at three different observation times (24 h, 7 days, and 28 days).

**Figure 6:**
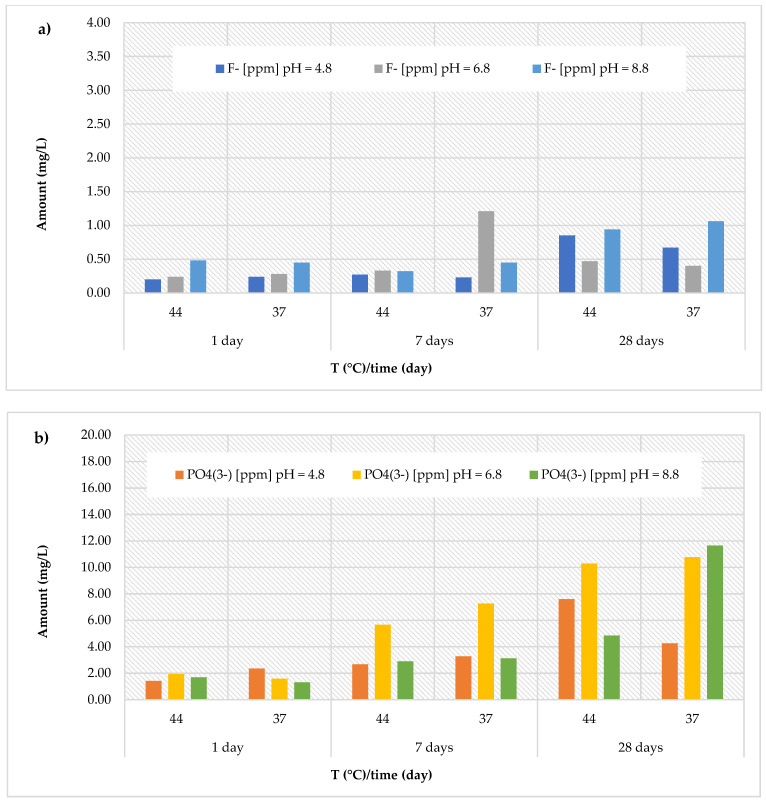
(**a**) Comparison of [F]^−^ release from *Cention Primer* material across three different pH levels, two temperatures (44 and 37 °C), and at three different observation times (24 h, 7 days, and 28 days); (**b**) comparison of [PO4^3−^] release from *Cention Primer* material across three different pH levels, two temperatures (44 and 37 °C), and at three different observation times (24 h, 7 days, and 28 days).

**Figure 7:**
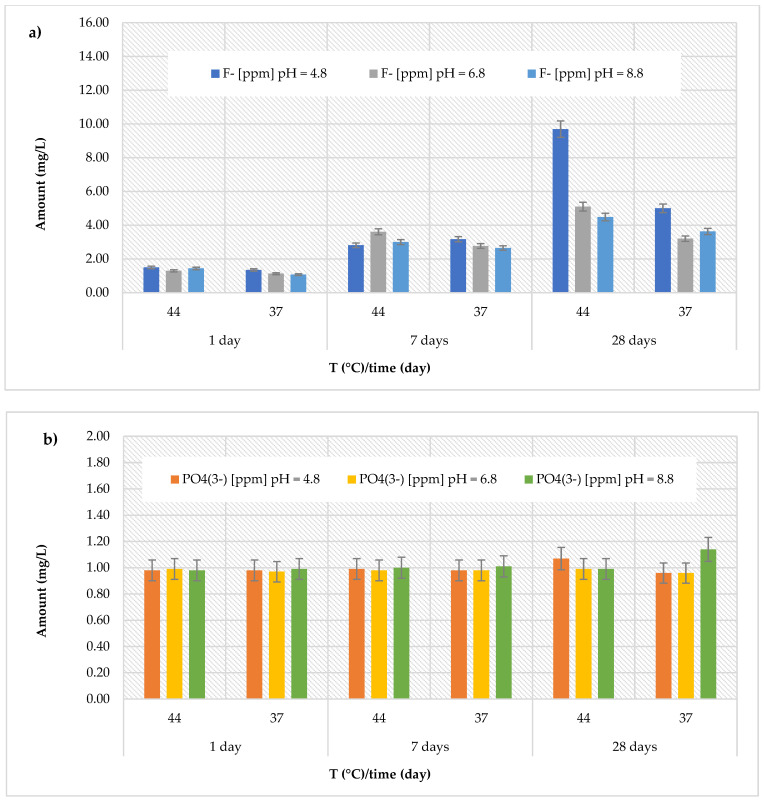
(**a**) Comparison of [F]^−^ release from *GC Fuji IX GP Fast* material across three different pH levels, two temperatures (44 and 37 °C), and at three different observation times (24 h, 7 days, and 28 days); (**b**) comparison of [PO_4_^3−^] release from *GC Fuji IX GP Fast* material across three different pH levels, two temperatures (44 and 37 °C), and at three different observation times (24 h, 7 days, and 28 days).

The releases of fluoride and phosphate ions obtained for all materials across three different pH levels, two temperatures (44 and 37 °C), and at three different observation times (24 h, 7 days, and 28 days) are showed in Figure 2, Figure 3, Figure 4, Figure 5, Figure 6 and Figure 7. Specifically, for the *Cention Forte Filling Material*, the release of fluoride ions showed an increasing trend with the observation times, temperature, and pH (Figure 2a). In fact, the highest concentration (11.25 ± 0.71 mg/L) was found after 28 days at 44 °C in the basic environment (pH = 8.8). The observed concentrations ranged from 0.85 ± 0.13 to 6.35 ± 0.22 mg/L at pH = 4.8 (average value 3.75 ± 2.20 mg/L), from 0.61 ± 0.07 to 8.85 ± 0.25 mg/L at pH = 6.8 (average value 3.54 ± 3.01 mg/L), and from 0.74 ± 0.09 to 11.25 ± 0.71 mg/L at pH = 8.8 (average value 4.87 ± 4.39 mg/L). Regarding the release of phosphate ions (Figure 2b), in the *Cention Forte Filling Material*, a nearly constant trend was observed, with values ranging from 0.95 ± 0.10 mg/L (at pH = 6.8 and T = 44 °C, after 7 days) to 1.17 ± 0.26 mg/L (at pH = 8.8 and T = 44 °C, after 28 days).

Similarly, for the *Stela Self Cure* material, the highest concentration of fluoride released (although much lower than the *Cention Forte Filling Material*) was found under the same conditions (Figure 3a). In fact, the highest concentrations (3.55 ± 0.24 mg/L) were found after 28 days at 44 °C, at pH = 8.8. Additionally, the recorded amount for this material ranged from 0.28 ± 0.07 to 2.82 ± 0.25 mg/L at pH = 4.8 (average value 0.89 ± 0.97 mg/L), from 0.22 ± 0.04 to 2.80 ± 0.26 mg/L at pH = 6.8 (average value 1.00 ± 0.96 mg/L), and from 0.23 ± 0.06 to 3.55 ± 0.24 mg/L at pH = 8.8 (average value 1.18 ± 1.23 mg/L). Regarding the release of phosphate ions (Figure 3b), like the *Cention Forte Filling Material*, in the *Stela Self Cure* material a constant trend was observed, with values ranging from 0.96 ± 0.12 mg/L (at pH = 8.8 and T = 44 °C, after 7 days) to 1.16 ± 0.13 mg/L (at pH = 6.8 and T = 37 °C, after 28 days).

The *Riva Light Cure HV* material showed higher amount than the *Cention Forte Filling Material*, but conversely, with the highest fluoride release recorded (40.14 ± 0.32 mg/L) after 28 days in acidic environment (pH = 4.8) at 44 °C (Figure 4a). Furthermore, the cumulative fluoride release ranged from 1.51 ± 0.25 to 40.14 ± 0.25 mg/L at pH = 4.8 (average value 10.43 ± 14.79 mg/L), from 2.04 ± 0.29 to 8.69 ± 0.33 mg/L at pH = 6.8 (average value 5.38 ± 2.96 mg/L), and from 2.63 ± 0.16 to 12.91 ± 0.44 mg/L at pH = 8.8 (average value 5.68 ± 3.79 mg/L). Regarding the release of phosphate ions (Figure 4b), the recorded values were higher than those observed for the previously discussed materials, ranging from 0.99 ± 0.12 mg/L (at pH = 8.8 and T = 37 °C, after 28 days) to 3.06 ± 0.19 mg/L (at pH = 8.8 and T = 44 °C, after 28 days). Similarly, the *Riva Self Cure* material indicated the highest fluoride release (7.67 ± 0.49 mg/L) after 28 days at 44 °C in the acid environment (pH = 4.8). The recorded amount ranged from 1.61 ± 0.31 to 7.67 ± 0.49 mg/L at pH = 4.8 (average value 4.25 ± 2.28 mg/L), from 1.41 ± 0.34 to 5.33 ± 0.47 mg/L at pH = 6.8 (average value 3.50 ± 1.71 mg/L), and from 1.29 ± 0.33 to 6.41 ± 0.44 mg/L at pH = 8.8 (average value 3.66 ± 1.88 mg/L). Regarding the release of phosphate ions (Figure 4b), the detected amount ranging from 0.90 ± 0.16 mg/L (at pH = 4.8 and T = 44 °C, after 7 days) to 1.12 ± 0.20 mg/L (at pH = 8.8 and T = 44 °C, after 28 days).

In contrast, for the *Equia Forte HT Fil*, the highest fluoride release (34.59 ± 0.63 mg/L) was observed after 28 days in basic environment (pH = 8.8) at 37 °C (Figure 5a). In particular, the cumulative fluoride release ranged from 1.27 ± 0.22 to 30.15 ± 0.43 mg/L at pH = 4.8 (average value 17.02 ± 13.10 mg/L), from 1.70 ± 0.34 to 17.30 ± 0.36 mg/L at pH = 6.8 (average value 10.78 ± 7.14 mg/L), and from 2.18 ± 0.23 to 34.59 ± 0.63 mg/L at pH = 8.8 (average value 19.32 ± 14.09 mg/L). Regarding the release of phosphate ions (Figure 5b), the cumulative release ranged from 0.91 ± 0.11 mg/L (at pH = 4.8 and T = 37 °C, after 28 days) to 1.06 ± 0.16 mg/L (at pH = 8.8 and T = 44 °C, after 28 days).

An opposite situation was observed for the *Cention Primer* material, which showed a lower release of fluoride ions (Figure 6a), but a much higher release of phosphate ions (Figure 6b) compared to all other materials. Specifically, the cumulative concentrations of fluoride ions recorded for this material ranged between 0.20 ± 0.02 and 0.85 ± 0.10 mg/L in an acidic environment (with an average value of 0.41 ± 0.28 mg/L), between 0.24 ± 0.03 and 1.21 ± 0.15 mg/L at neutral pH (with an average value of 0.49 ± 0.36 mg/L), and between 0.32 ± 0.04 and 1.06 ± 0.13 mg/L at basic pH (with an average value of 0.62 ± 0.30 mg/L). Interestingly, the release of phosphate ions was remarkably high, with the maximum value (11.64 ± 1.40 mg/L) observed after 28 days at 37 °C in a basic environment (pH = 8.8). Specifically, the cumulative concentrations of phosphate ions ranged from 1.42 ± 0.17 to 7.60 ± 0.91 mg/L (at pH = 4.8, with an average value of 3.60 ± 2.18 mg/L), from 1.58 ± 0.19 to 10.77 ± 1.29 mg/L (at pH = 6.8, with an average value of 6.25 ± 3.97 mg/L), and from 1.32 ± 0.16 to 11.64 ± 1.40 mg/L (at pH = 8.8, with an average value of 4.26 ± 3.83 mg/L).

Finally, for the *GC Fuji IX GP Fast* material, the highest fluoride concentrations (9.69 ± 0.57 mg/L) were detected in an acidic environment at 44 °C after 28 days (Figure 7a). Specifically, the cumulative concentrations of fluoride ions ranged between 1.35 ± 0.26 and 9.69 ± 0.57 mg/L (with an average value of 3.92 ± 3.12 mg/L in an acidic environment), between 1.13 ± 0.19 and 5.10 ± 0.25 mg/L (with an average value of 2.85 ± 1.49 mg/L in a neutral environment), and between 1.07 ± 0.14 and 4.48 ± 0.28 mg/L (with an average value of 2.71 ± 1.29 mg/L in a basic environment). Regarding the release of phosphate ions (Figure 7b), a consistent trend was observed, with concentrations ranging between 0.96 ± 0.05 and 1.14 ± 0.15 mg/L.

In summary, the release behaviors of fluoride and phosphate ions across the evaluated materials revealed distinct patterns influenced by pH, temperature, and observation time. While some materials, such as the *Cention Forte Filling*, demonstrated a marked increase in fluoride ion release with higher temperatures and pH levels, others, like the Riva Light Cure and *Equia Forte HT Fit*, exhibited higher fluoride release in acidic or basic environments. Conversely, phosphate ion release generally displayed a more constant trend, with notable exceptions like the *Cention Primer*, which showed significantly elevated phosphate release compared to all other materials. These findings underscore the diverse release profiles of the tested materials, highlighting their potential variability in clinical applications depending on the surrounding environment and conditions.

### 3.3. Release of Calcium, Silicon, and Strontium

**Figure 8:**
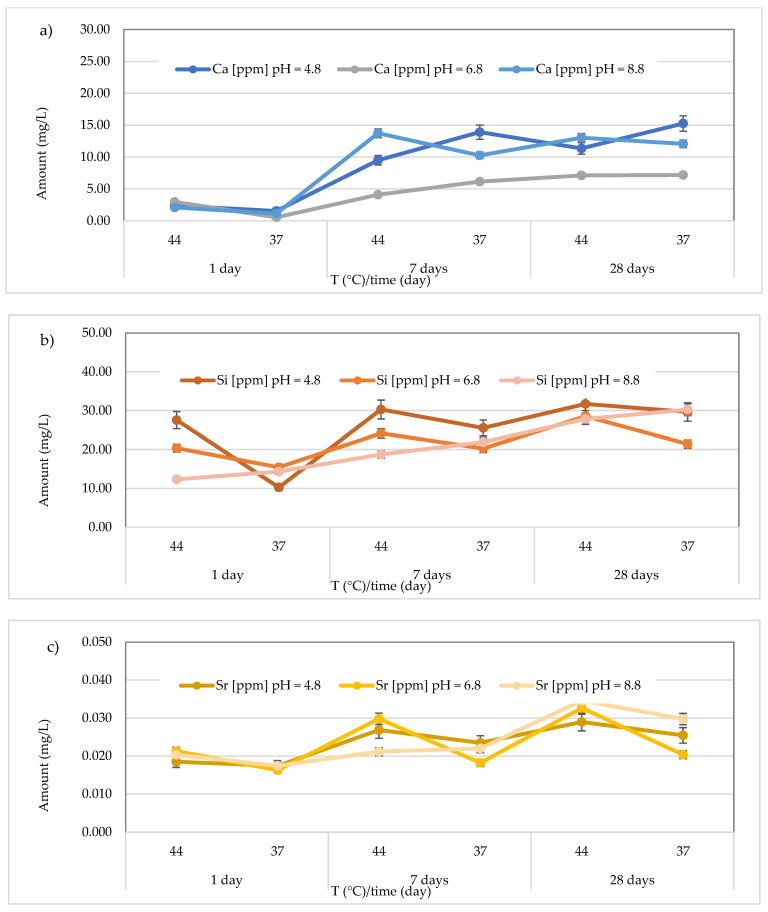
(**a**) Comparison of Ca^2+^ release from *Cention Forte Filling Material* across three different pH levels, two temperatures (44 and 37 °C), and at three different observation times (24 h, 7 days, and 28 days); (**b**) comparison of Si release from *Cention Forte Filling Material* across three different pH levels, two temperatures (44 and 37 °C), and at three different observation times (24 h, 7 days, and 28 days); (**c**) comparison of Sr release from *Cention Forte Filling Material* across three different pH levels, two temperatures (44 and 37 °C), and at three different observation times (24 h, 7 days, and 28 days).

**Figure 9:**
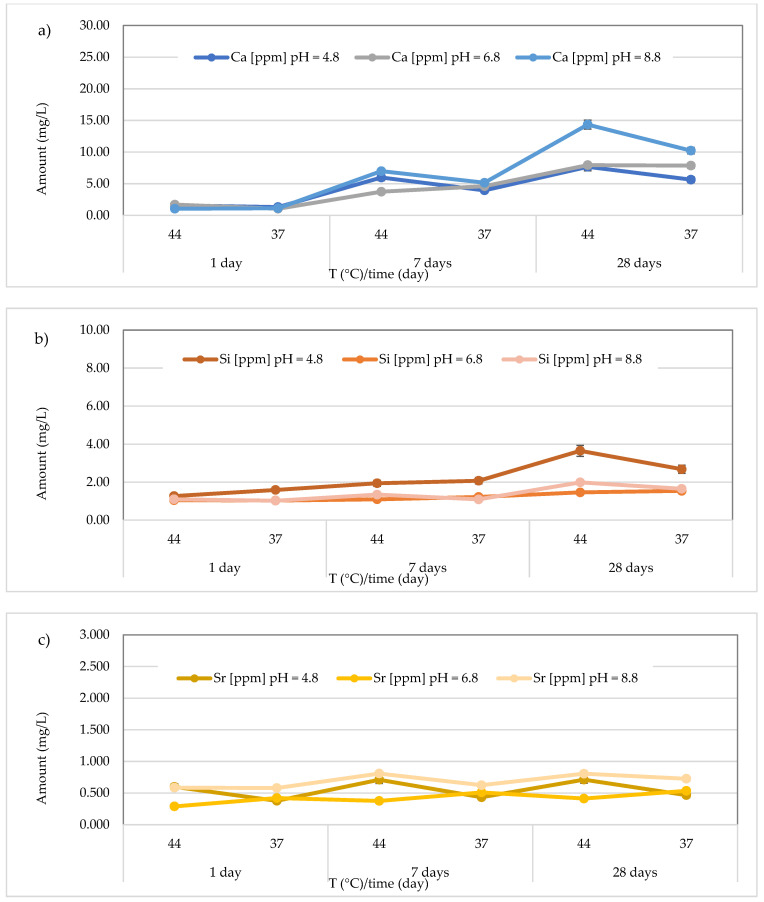
(**a**) Comparison of Ca^2+^ release from *Stela Self Cure* material across three different pH levels, two temperatures (44 and 37 °C), and at three different observation times (24 h, 7 days, and 28 days); (**b**) comparison of Si release from *Stela Self Cure* material across three different pH levels, two temperatures (44 and 37 °C), and at three different observation times (24 h, 7 days, and 28 days); (**c**) comparison of Sr release from *Stela Self Cure* material across three different pH levels, two temperatures (44 and 37 °C), and at three different observation times (24 h, 7 days, and 28 days).

**Figure 10:**
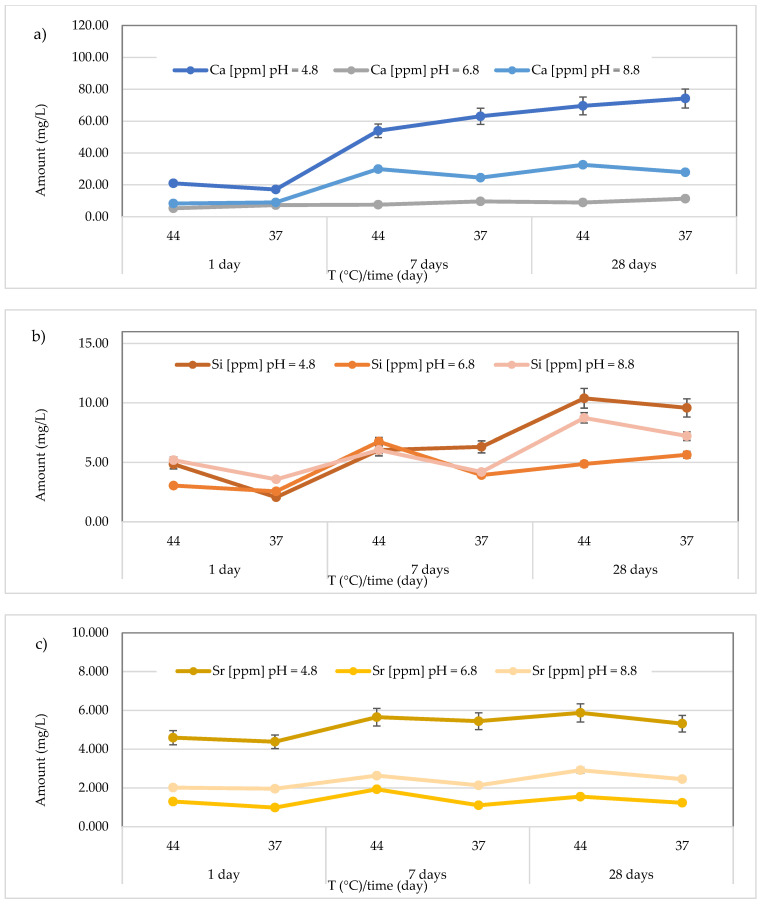
(**a**) Comparison of Ca^2+^ release from *Riva Light Cure* material across three different pH levels, two temperatures (44 and 37 °C), and at three different observation times (24 h, 7 days, and 28 days); (**b**) comparison of Si release from *Riva Light Cure* material across three different pH levels, two temperatures (44 and 37 °C), and at three different observation times (24 h, 7 days, and 28 days); (**c**) comparison of Sr release from *Riva Light Cure* material across three different pH levels, two temperatures (44 and 37 °C), and at three different observation times (24 h, 7 days, and 28 days).

**Figure 11:**
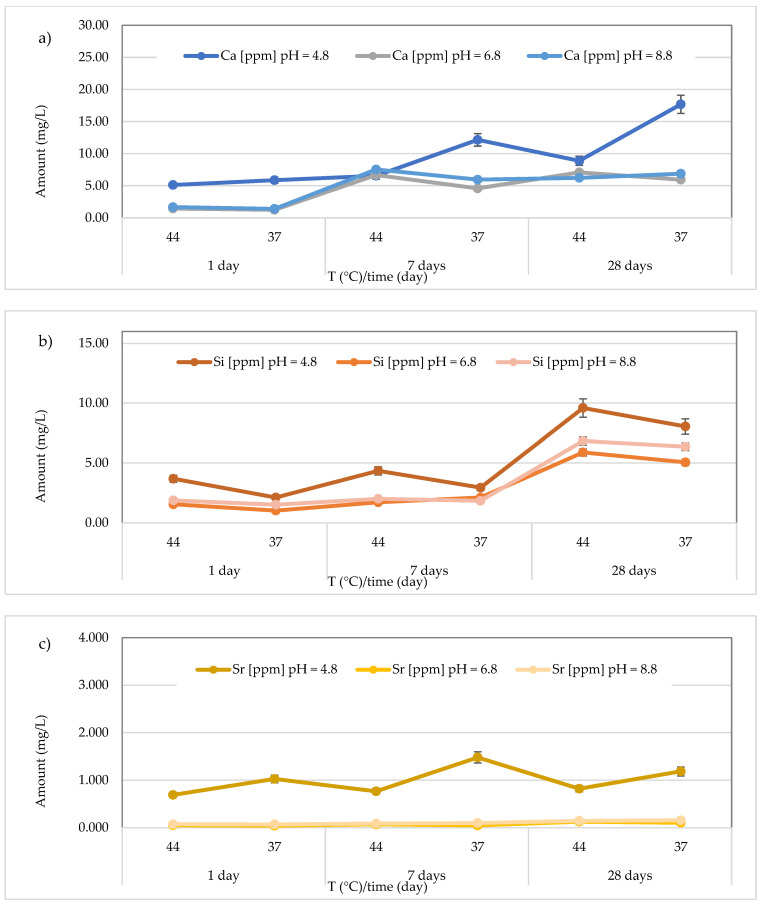
(**a**) Comparison of Ca^2+^ release from *Riva Self Cure* material across three different pH levels, two temperatures (44 and 37 °C), and at three different observation times (24 h, 7 days, and 28 days); (**b**) comparison of Si release from *Riva Self Cure* material across three different pH levels, two temperatures (44 and 37 °C), and at three different observation times (24 h, 7 days, and 28 days); (**c**) comparison of Sr release from *Riva Self Cure* material across three different pH levels, two temperatures (44 and 37 °C), and at three different observation times (24 h, 7 days, and 28 days).

**Figure 12:**
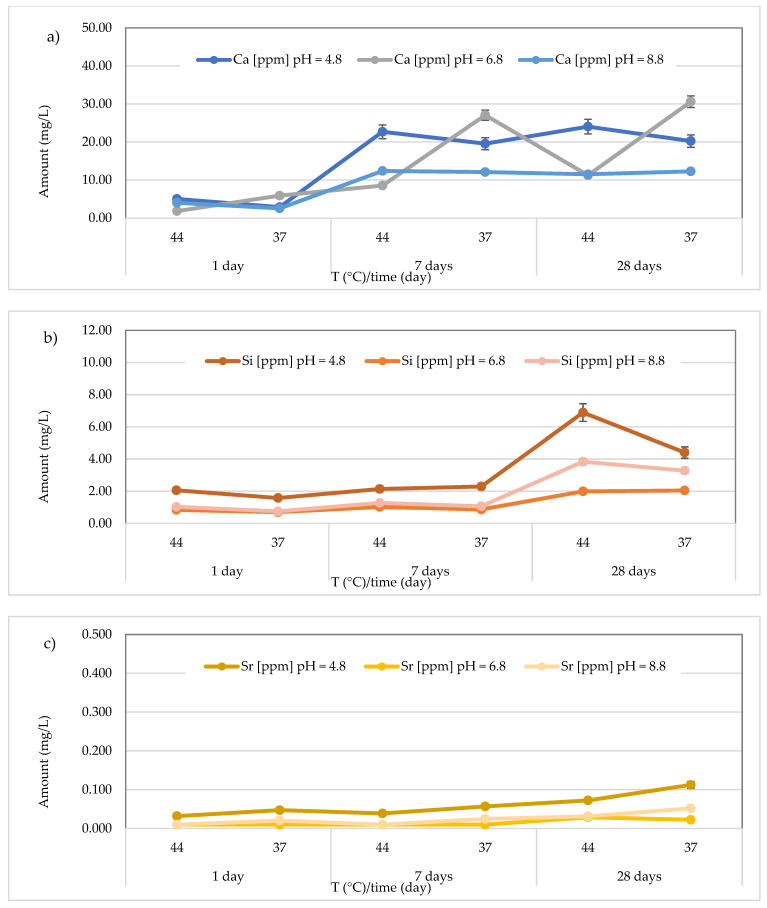
(**a**) Comparison of Ca^2+^ release from *Equia Forte HT Fil* material across three different pH levels, two temperatures (44 and 37 °C), and at three different observation times (24 h, 7 days, and 28 days); (**b**) comparison of Si release from *Equia Forte HT Fil* material across three different pH levels, two temperatures (44 and 37 °C), and at three different observation times (24 h, 7 days, and 28 days); (**c**) comparison of Sr release from *Equia Forte HT Fil* material across three different pH levels, two temperatures (44 and 37 °C), and at three different observation times (24 h, 7 days, and 28 days).

**Figure 13:**
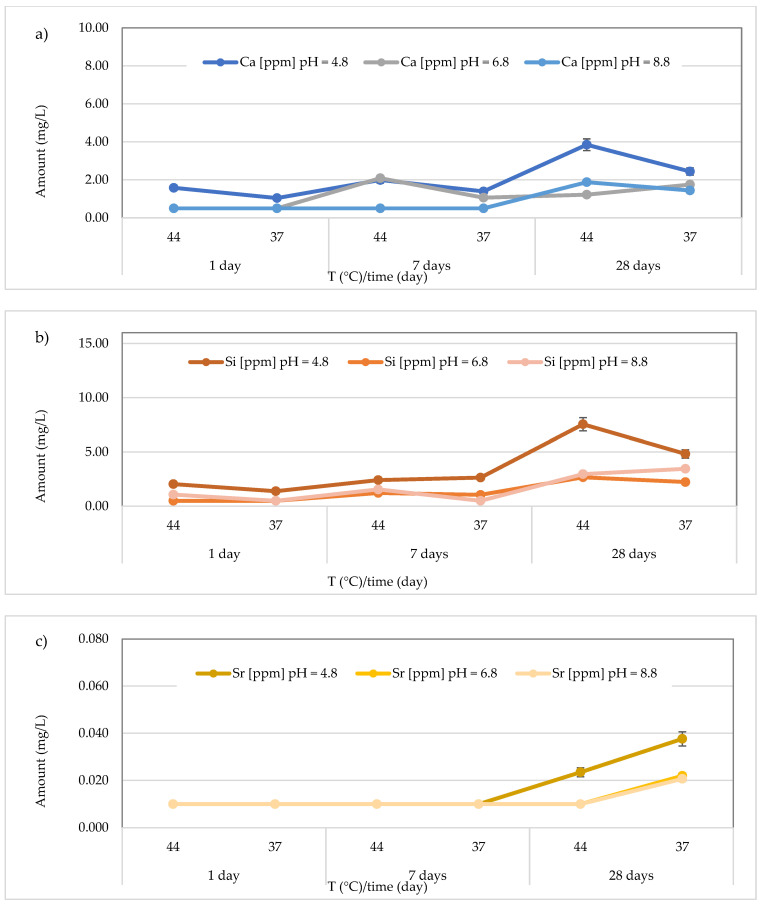
(**a**) Comparison of Ca^2+^ release from *Cention Primer* material across three different pH levels, two temperatures (44 and 37 °C), and at three different observation times (24 h, 7 days, and 28 days); (**b**) comparison of Si release from *Cention Primer* material across three different pH levels, two temperatures (44 and 37 °C), and at three different observation times (24 h, 7 days, and 28 days); (**c**) comparison of Sr release from *Cention Primer* material across three different pH levels, two temperatures (44 and 37 °C), and at three different observation times (24 h, 7 days, and 28 days).

**Figure 14:**
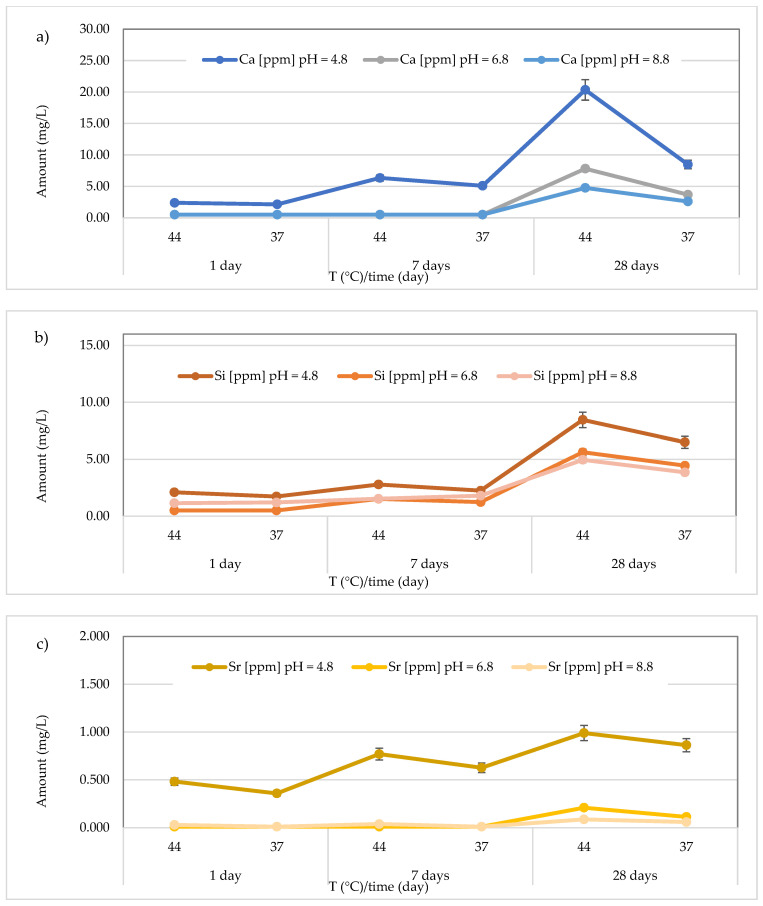
(**a**) Comparison of Ca^2+^ release from *GC Fuji IX GP Fast* material across three different pH levels, two temperatures (44 and 37 °C), and at three different observation times (24 h, 7 days, and 28 days); (**b**) comparison of Si release from *GC Fuji IX GP Fast* material across three different pH levels, two temperatures (44 and 37 °C), and at three different observation times (24 h, 7 days, and 28 days); (**c**) comparison of Sr release from *GC Fuji IX GP Fast* material across three different pH levels, two temperatures (44 and 37 °C), and at three different observation times (24 h, 7 days, and 28 days).

The results concerning the release of calcium, silicon, and strontium for all materials across three different pH levels, two temperatures (44 and 37 °C), and three different observation times (24 h, 7 days, and 28 days) are presented in Figure 8, Figure 9, Figure 10, Figure 11, Figure 12, Figure 13 and Figure 14.

Regarding Ca^2+^ release, for the *Cention Forte Filling Material*, the highest concentration (15.26 ± 0.45 mg/L) was found after 28 days at 37 °C in the acid environment (pH = 4.8). Specifically, the observed concentrations ranged from 1.556 ± 0.15 to 15.26 ± 0.45 mg/L at pH = 4.8 (average value 9.00 ± 5.80 mg/L), from 0.56 < 1.0 to 7.20 ± 0.23 mg/L at pH = 6.8 (average value 4.69 ± 2.64 mg/L), and from 1.15 ± 0.23 to 13.75 ± 0.28 mg/L at pH = 8.8 (average value 8.73 ± 5.63 mg/L) (Figure 8a). In the same material, the release of Si was also observed, and the highest concentration was detected at 37 °C after 28 days in an acidic environment. Specifically, the concentrations ranged between 10.24 ± 0.42 and 31.72 ± 0.68 mg/L (mean value 25.83 ± 7.94 mg/L) in the acidic environment, 15.44 ± 0.33 to 28.56 ± 0.40 mg/L (mean value 21.69 ± 4.39 mg/L) in the neutral environment, and 12.33 ± 0.36 to 30.25 ± 0.47 mg/L (mean value 20.89 ± 7.19 mg/L) in the basic environment (Figure 8b). Additionally, for Sr release from *Cention Forte Filling Material*, shown in Figure 8c, the highest concentration was observed in a basic environment at 44 °C after 28 days (0.035 ± 0.008 mg/L). Specifically, Sr concentrations released from the material ranged from 0.017 ± 0.003 to 0.029 ± 0.006 mg/L (mean value 0.023 ± 0.005 mg/L) in the acidic environment, 0.016 ± 0.001 to 0.033 ± 0.006 mg/L (mean value 0.023 ± 0.007 mg/L) in the neutral environment, and 0.017 ± 0.001 to 0.035 ± 0.008 mg/L in the basic environment, with a mean value of 0.024 ± 0.007 mg/L. Regarding Sb, no release was observed from *Cention Forte Filling Material*, as all concentrations were below the LOQ.

In the Stela Self Cure material, the highest Ca^2+^ release (14.35 ± 0.45 mg/L), in contrast to what was observed for *Cention Forte Filling Material*, was detected after 28 days at 44 °C in the basic environment (pH = 8.8). In detail, the found concentrations ranged from 1.31 ± 0.28 to 7.68 ± 0.29 mg/L at pH = 4.8 (average value 4.35 ± 2.55 mg/L), from 1.05 ± 0.05 to 7.94 ± 0.25 mg/L at pH = 6.8 (average value 4.48 ± 2.95 mg/L), and from 1.04 ± 0.11 to 14.35 ± 0.45 mg/L at pH = 8.8 (average value 6.48 ± 5.22 mg/L) (Figure 9a). In the same material, the release of Si was also observed, and the highest concentration was detected at 44 °C after 28 days in an acidic environment. Specifically, the concentrations ranged between 1.27 ± 0.17 and 3.65 ± 0.19 mg/L (mean value 2.20 ± 0.86 mg/L) in the acidic environment, from 1.03 ± 0.11 to 1.54 ± 0.25 mg/L (mean value 1.23 ± 0.22 mg/L) in the neutral environment, and 1.02 ± 0.11 to 1.99 ± 0.24 mg/L (mean value 1.37 ± 0.38 mg/L) in the basic environment (Figure 9b). Additionally, for Sr release from the material *Stela Self Cure*, shown in Figure 9c, the highest concentration was observed in a basic environment at 44 °C after 7 days (0.808 ± 0.017 mg/L), a value similar to that observed at the same temperature and pH after 28 days (0.805 ± 0.036 mg/L). Furthermore, Sr concentrations released from the material ranged from 0.378 ± 0.033 to 0.712 ± 0.015 mg/L (mean value 0.55 ± 0.14 mg/L) in the acidic environment, 0.290 ± 0.035 to 0.5364 ± 0.022 mg/L (mean value 0.42 ± 0.09 mg/L) in the neutral environment, and 0.580 ± 0.016 to 0.8081 ± 0.017 mg/L in the basic environment, with a mean value of 0.69 ± 0.11 mg/L.

Figure 10 illustrates the results obtained for the *Riva Light Cure* material. The highest Ca^2+^ release (74.23 ± 0.37 mg/L) was found after 28 days at 37 °C in the acidic media (pH = 4.8). Additionally, the detected concentrations ranged from 17.12 ± 0.18 to 74.23 ± 0.37 mg/L at pH = 4.8 (average value 49.81 ± 24.82 mg/L), from 5.31 ± 0.27 to 11.36 ± 0.50 mg/L at pH = 6.8 (average value 8.35 ± 2.10 mg/L), and from 8.32 ± 0.62 to 32.58 ± 0.62 mg/L at pH = 8.8 (average value 22.04 ± 10.70 mg/L) (Figure 10a). In the same material, the release of Si was also observed, and the highest concentration (10.39 ± 0.62 mg/L) was detected at 44 °C after 28 days in an acidic environment. Specifically, the concentrations ranged between 2.07 ± 0.20 and 10.39 ± 0.69 mg/L (mean value 6.54 ± 3.07 mg/L) in the acidic environment, 2.57 ± 0.21 to 6.75 ± 0.26 mg/L (mean value 4.47 ± 1.59 mg/L) in the neutral environment, and 3.58 ± 0.37 to 8.74 ± 0.37 mg/L (mean value 5.83 ± 1.93 mg/L) in the basic environment (Figure 10b). Additionally, for Sr release from the material *Riva Light Cure* material, shown in Figure 10c, the highest concentration was observed in the acidic environment at 44 °C after 28 days (5.87 ± 0.06 mg/L). Furthermore, Sr concentrations released from the material ranged from 4.38 ± 0.07 to 5.87 ± 0.07 mg/L (mean value 5.21 ± 0.59 mg/L) in the acidic environment, 0.99 ± 0.08 to 1.93 ± 0.05 mg/L (mean value 1.35 ± 0.34 mg/L) in the neutral environment, and 1.96 ± 0.07 to 2.91 ± 0.14 mg/L in the basic environment, with a mean value of 2.35 ± 0.38 mg/L.

In the *Riva Self Cure* material, the Ca^2+^ amount released was in range 5.12 ± 0.21–17.69 ± 0.42 mg/L, with a mean value of 9.38 ± 4.81 mg/L at pH = 4.8, 1.25 ± 0.32–7.08 ± 0.23 mg/L, with a mean value of 4.49 ± 2.58 mg/L at pH = 6.8 and 1.40 ± 0.38 to 7.54 ± 0.43 mg/L, with a mean value of 4.94 ± 2.70 mg/L at pH = 8.8. Therefore, the highest release was found in the acidic environment at 37 °C after 28 days (Figure 11a). Regarding Si release, the highest amount was found in the acidic environment after 28 days at 44 °C (9.60 ± 0.40 mg/L) (Figure 11b). Moreover, the amount of Si ranged from 2.12 ± 0.28 to 9.60 ± 0.40 mg/L (mean value 5.13 ± 3.00 mg/L) at pH = 4.8, from 1.03 ± 0.19 to 5.89 ± 0.20 mg/L (mean value 2.89 ± 2.05 mg/L) at pH = 6.8 and from 1.52 ± 0.32 to 6.85 ± 0.19 mg/L (mean value 3.41 ± 2.49 mg/L) at pH = 8.8. Additionally, for Sr release from the material *Riva Self Cure* material (Figure 11c), the highest concentration was observed in the acidic environment at 37 °C after 7 days (1.48 ± 0.08 mg/L). However, the Sr amount ranged from 0.69 ± 0.04 to 1.48 ± 0.08 mg/L (mean value 0.99 ± 0.30 mg/L) in the acidic environment, 0.042 ± 0.012 to 0.125 ± 0.022 mg/L (mean value 0.072 ± 0.034 mg/L) in the neutral environment, and 0.070 ± 0.010 to 0.156 ± 0.017 mg/L in the basic environment, with a mean value of 0.11 ± 0.04 mg/L.

Regarding the material *Equia Forte HT Fil*, the highest concentrations of Ca^2+^ were observed in the neutral medium (pH 6.8), with a maximum value of 30.60 ± 0.73 mg/L after 28 days at 37 °C (Figure 12a). The concentrations had a mean value of 15.74 ± 9.29 mg/L (range from 2.89 ± 0.22 to 24.05 ± 0.26 mg/L) in the acidic environment, 14.20 ± 11.81 mg/L (range from 1.85 ± 0.16 to 30.60 ± 0.73 mg/L) in the neutral environment, and 9.14 ± 4.56 mg/L (range from 2.56 ± 0.25 to 12.39 ± 0.47 mg/L) in the basic environment. About the Si, the highest amount was found in the acidic environment after 28 days at 44 °C (6.89 ± 0.25 mg/L) (Figure 12b). Moreover, the amount of Si ranged from 1.58 ± 0.27 to 6.89 ± 0.25 mg/L (mean value 3.23 ± 2.05 mg/L) at pH = 4.8, from 0.70 ± 0.09 to 2.05 ± 0.17 mg/L (mean value 1.24 ± 0.61 mg/L) at pH = 6.8 and from 0.75 ± 0.13 to 3.84 ± 0.27 mg/L (mean value 1.87 ± 1.33 mg/L) at pH = 8.8.

As observed in Figure 12c, the *Equia Forte HT Fil* material showed Sr concentrations that were almost similar under all conditions, with a maximum value of 0.112 ± 0.013 mg/L, detected after 28 days in the acidic environment at 37 °C. For the *Cention Primer* material, the concentrations detected for all the studied metals were lower compared to those obtained for other materials. Specifically, for calcium, the concentrations ranged between 1.04 ± 0.12 and 3.85 ± 0.46 mg/L at acidic pH, <LOQ and 2.09 ± 0.25 mg/L at neutral pH, and <LOQ and 1.88 ± 0.23 mg/L at basic pH, with mean values of 2.05 ± 1.01 mg/L, 1.19 ± 0.65 mg/L, and 0.89 ± 0.62 mg/L, respectively (Figure 13a). The maximum value was found after 28 days at 44 °C in an acidic environment (3.85 ± 0.46 mg/L). In the same material, the concentrations of Si obtained were in the ranges of 1.39 ± 0.17 to 7.55 ± 0.91 mg/L, <LOQ to 2.67 ± 0.32 mg/L, and <LOQ to 3.45 ± 0.41 mg/L in acidic, neutral, and basic environments, respectively. Notably, the highest release of Si was observed in an acidic environment at 44 °C after 28 days (Figure 13b). The Sr concentrations are illustrated in Figure 13c, showing that the highest concentrations were found in an acidic environment (0.038 ± 0.005 mg/L at 37 °C after 28 days).

Finally, for the *GC Fuji IX GP Fast* material, the results are shown in Figure 14. Specifically, regarding calcium release, the concentrations ranged between 2.14 ± 0.28 and 20.35 ± 0.47 mg/L (mean value 7.46 ± 6.75 mg/L) in an acidic environment, <LOQ to 7.82 ± 0.24 mg/L (mean value 2.25 ± 3.01 mg/L) in a neutral environment, and <LOQ to 4.77 ± 0.23 mg/L (mean value 1.56 ± 1.78 mg/L) in a basic environment. The maximum value was observed after 28 days in an acidic environment at 44 °C (Figure 14a). For Si, the highest concentration detected was 8.45 ± 0.36 mg/L (at 44 °C, pH 4.8, after 28 days) (Figure 14b). Similarly, for Sr, the highest concentration was found under the same conditions (0.990 ± 0.035 mg/L) (Figure 14c).

In conclusion, the analysis of the metal ion release from the various dental materials reveals significant differences in their release profiles. Materials such as *Cention Forte Filling Material* and *Stela Self Cure* showed notable variation in metal release, with *Cention Forte Filling Material* exhibiting higher calcium and silicon concentrations, particularly in acidic environments, while *Stela Self Cure* material demonstrated the highest release of calcium in basic conditions. Conversely, materials like *Cention Primer* and *Equia Forte HT Fil* displayed much lower concentrations of metal release across all conditions, with *Cention Primer* showing consistently low calcium and silicon release, and *Equia Forte HT Fil* presenting minimal strontium release. These observations highlight the varied release behavior of different dental materials, emphasizing the importance of selecting materials based on specific clinical requirements and the potential impact on the oral environment over time.

Looking at the present results and according to previous investigations [30], the most significant failures or weaknesses in the materials were observed under acidic conditions (pH 4.8) and high temperatures (44 °C), where ion release was highest. Specifically, Riva Light Cure HV exhibited the highest fluoride (40.14 mg/L) and calcium (74.23 mg/L) release under acidic conditions at 44 °C after 28 days. This suggests potential degradation or faster wear in acidic environments. Strontium release was also highest in Riva Light Cure HV (5.87 mg/L) under these extreme conditions, indicating that this material might be more affected by acidic conditions. Cention Forte Filling had the highest silicon release (31.72 mg/L) at 37 °C under acidic conditions, which could imply structural breakdown. Regarding feasibility, materials that more consistently released ions over time without extreme fluctuations might be more suitable for long-term use. Equia Forte HT Fil showed relatively lower ion release across different conditions, suggesting better stability. If the goal is high remineralization potential, Riva Light Cure HV may be favorable, but it might degrade more under highly acidic conditions. If long-term stability is the priority, materials with slower overall ion release, like Equia Forte HT Fil, may be more suitable [31].

### 3.4. Results of the Statistical Analysis

The results of the statistical analysis indicated that the release of the studied ions is significantly influenced by the considered factors, namely the acidity conditions of the medium, temperature, and exposure time, with different trends observed across the tested materials.

Specifically, fluoride release was significantly affected by pH (*p* = 0.009) and exposure time (*p* < 0.001), with higher fluoride concentrations generally observed under acidity stress conditions, either at acidic or basic pH levels, and over prolonged observation periods. Calcium showed a negative correlation with pH (*p* < 0.001), suggesting greater solubilization under acidic conditions, while time had a significant positive effect (*p* < 0.001), indicating sustained release over time. Silicon release exhibited statistically significant effects for all tested parameters, with pH (*p* < 0.001), temperature (*p* < 0.001), and time (*p* < 0.001) playing key roles, indicating that its release is influenced by multiple physicochemical factors. Strontium release was significantly affected by pH (*p* < 0.001), while temperature (*p* = 0.424) and exposure time (*p* = 0.432) did not show significant effects, suggesting lower susceptibility to temperature and duration changes. Lastly, phosphate release showed a significant dependence on time (*p* < 0.001), while pH and temperature had no statistically relevant effects (*p* > 0.05), suggesting greater stability compared to other ions.

Furthermore, the multivariate analysis confirmed the crucial role of exposure time and pH in regulating ion release, with significant differences observed among the tested materials (*p* < 0.05). These findings highlight the importance of considering environ-mental conditions when selecting restorative materials, as their behavior may vary in response to changes in pH and temperature within the oral cavity.

## 4. Discussion

Dental caries is a biofilm-mediated, diet-modulated, multifactorial, dynamic disease that results in a progressive decrease in dental hard tissue [7]. Secondary caries may develop due to increased bacterial adhesion and biofilm development, especially on proximal surfaces with resin composite restorations where plaque control is difficult [32]. It has also been amply demonstrated in the literature that polymerization shrinkage and masticatory load can lead to high levels of stress, especially in deep class I cavities made of resin-based composite materials, favoring the development of secondary caries and dental hypersensitivity [33,34]. Since some resin-based composites can promote bacterial growth around them, various non-invasive or micro-invasive strategies have been proposed, including the use of bioactive restorative materials capable of releasing ions, especially fluoride and calcium, that can reduce the adhesion and proliferation of oral bacteria on their surfaces, resulting in less plaque accumulation [35].

Bioactive restorative materials are a new development in dentistry; they maintain dental health and function through biological actions related to their antimicrobial properties, such as reducing biofilm activity, preventing demineralization of surrounding tissues, and promoting remineralization of caries-affected areas [36]. Bioactive restorative materials also release and recharge ionic components in response to changes in pH and are moisture resistant, allowing continuous ion exchange with oral fluid [37].

The primary goal of this study was to investigate how pH and temperature affect the release of ions such as Ca^2+^, F^−^, PO_4_^3−^, OH^−^, Si, and Sr^2+^ from various bioactive polymers or acid-base dental filling materials. This is crucial because ion release plays a central role in promoting remineralization and enhancing the biological compatibility of dental restorations. The study measured ion release over varying conditions using advanced techniques like ion chromatography and mass spectrometry, which enabled the precise quantification of the ion concentrations over 28 days. The results confirmed that both pH and temperature significantly influence ion release, which, probably, in turn impacts the biological interactions and functional longevity of bioactive materials in dental applications.

The pH of the oral environment is a major determinant of the solubility and ion release from bioactive materials. Acidic conditions in the oral cavity, often resulting from the consumption of acidic foods and beverages, trigger an increased release of ions such as Ca^2+^, F^−^, and PO_4_^3−^. This study revealed that pH had a significant impact on ion release across all materials tested. Materials exposed to an acidic environment (pH 4.8) generally showed the highest ion release, particularly fluoride and calcium, which are key components in the remineralization of enamel. The observed peak ion releases at pH 4.8, especially in *Riva Light Cure HV*, suggest that acidic conditions enhance the dissolution of material components, promoting ion release. This is consistent with previous studies, such as those by Melo et al. 2023 [38], which found that acidic conditions increase the solubility of materials containing fluoride and calcium, resulting in higher ion concentrations in the solution. In the case of *Riva Light Cure HV*, the significant fluoride release (40.14 ± 0.32 mg/L) and calcium release (74.23 ± 0.37 mg/L) observed at pH 4.8 may be beneficial for caries prevention, as these ions are integral to enamel remineralization. Similarly, the release of strontium (5.87 ± 0.06 mg/L) in these conditions aligns with the role of strontium in promoting remineralization and strengthening the structure of dental hard tissues, which is especially relevant under acidic conditions that typically lead to enamel demineralization.

Temperature is another critical factor influencing the release of ions from bioactive dental materials. According to Madi et al. 2020 [39], an increase in temperature generally results in a higher release rate of ions, including Ca^2+^, F^−^, PO_4_^3−^, Sr^2+^, and Si. This is because elevated temperatures promote the dissolution of the material’s constituent compounds. Specifically, higher temperatures accelerate the solubility of silicate, phosphate, fluoride, and calcium compounds, facilitating the release of these ions. This phenomenon is particularly relevant in the oral cavity, where the temperature fluctuates during the consumption of hot foods and beverages. Specifically, 44 °C was chosen to simulate extreme but plausible thermal conditions that could potentially influence the behavior of dental restorative materials. This temperature helps to accelerate the material’s ion release process, mimicking a worst-case scenario for material degradation and ion leaching. However, it is well documented that temperatures can briefly exceed 37 °C in certain clinical situations, especially following the ingestion of hot food or beverages, or when patients undergo restorative treatments.

Moreover, elevated temperatures could also contribute to a more rapid ion exchange process, potentially enhancing the therapeutic effect of bioactive materials in promoting remineralization. This was particularly noticeable in *Riva Light Cure HV*, where the highest fluoride and calcium release was observed at both low pH (4.8) and higher temperature (44 °C). The combined influence of acidic pH and elevated temperature (44 °C) on *Riva Light Cure HV* suggests that these conditions could mimic the oral environment during certain physiological conditions (e.g., the consumption of hot, acidic beverages), where ion release is maximized. This characteristic may be beneficial for patients at higher risk of caries or enamel demineralization, as the material is actively releasing fluoride and calcium to facilitate remineralization.

Strontium release, which was highest in *Riva Light Cure HV* under acidic pH (4.8) and high temperature (44 °C), is noteworthy. Strontium is known to enhance the remineralization process by substituting calcium in hydroxyapatite crystals, increasing their resistance to acid dissolution. The release of strontium, especially under conditions that mimic the oral cavity during acidic or high-temperature states, is likely to contribute positively to the long-term protection of dental tissues [40,41,42]. These findings support the inclusion of strontium in bioactive dental materials, particularly for patients with a high caries risk or those undergoing restorative treatments.

Interestingly, *Cention Forte Filling Material* showed the highest silicon release (31.72 ± 0.68 mg/L) under the conditions of pH 4.8 and 37 °C. Silicon is an important component in bioactive materials due to its role in stimulating hydroxyapatite formation and promoting tissue regeneration [43]. The release of Si from *Cention Forte Filling Material* under acidic conditions could be advantageous for the long-term repair of dental tissues, as silicon promotes the formation of a mineralized layer on the material’s surface, enhancing its bioactivity and biocompatibility. This finding underscores the significance of silicon in bioactive dental materials. The temperature and pH conditions at which this release occurs are relevant to the oral environment, where acidic conditions often lead to demineralization and where the release of silicon could enhance the repair of dental tissues.

Phosphate (PO_4_^3−^) and hydroxide (OH^−^) ions are essential for the formation of hydroxyapatite, the primary mineral found in teeth and bones. Specifically, phosphate ions are critical in the reformation of hydroxyapatite crystals on the surface of the material, while hydroxide ions contribute to the stabilization of these crystals. As noted by Par et al. 2022, the release of PO_4_^3−^ and OH^−^ ions is particularly high in bioactive materials like glass ionomer cements, which are known for their ability to bond chemically to dental tissues and release fluoride, phosphate, and hydroxide ions in response to acidic conditions [43]. Under acidic conditions, the increased availability of PO_4_^3−^ and OH^−^ ions promote the deposition of remineralized hydroxyapatite, contributing to the restoration of enamel hardness and preventing further demineralization. However, the excessive release of OH^−^ ions at high temperatures may lead to undesirable effects, such as material degradation and alteration of the pH balance within the oral cavity.

Finally, this in vitro study also measured ion release over different time points: 1 day, 7 days, and 28 days. In the context of this study, 28 days was selected based on previous research of di Lauro et al., 2023 [30] and industry standards for simulating long-term exposure in vitro. In dental restorations, materials are expected to perform over extended periods, but practical considerations often limit the duration of in vitro studies. While 28 days is not the same as decades of real-world use, it is commonly regarded as an effective surrogate for accelerated aging and short-to-medium term exposure under controlled laboratory conditions, mainly for monomers and polymers dental materials. After curing, in fact, within 24 h, these materials release a very low percentage of ions in subsequent days, as also shown in the present study, because of the cross-linked chains that consistently limit the ion-releasing reaction of the residual acid-base reaction of the material itself. This period (28 days) allows us to observe trends in ion release and material behavior while minimizing the complexities of even longer exposure times.

The results showed a progressive release of ions over time, which suggests that bioactive materials continue to release beneficial ions in the long term. This sustained release is essential for maintaining a therapeutic effect, as continuous ion release can provide prolonged protection against demineralization and support ongoing remineralization of the tooth structure.

For instance, *Riva Light Cure HV* continued to release significant amounts of fluoride, calcium, and strontium over the 28-day period, reinforcing the material’s potential for long-term effectiveness in preventing caries and promoting remineralization. These results align with the general properties of bioactive materials, which are designed to release ions gradually to enhance their therapeutic effect over time.

Although this study provides valuable data on ion release from bioactive materials, there are limitations. For example, the study was conducted in controlled laboratory conditions, which may not fully replicate the complexity of the oral environment, where factors like salivary flow, pH fluctuations, and dietary habits such as commercial acidic drinks can influence the behavior of restorative materials, mainly in scholastic population where the daily use is potentially dangerous for a slow enamel demineralization in the time. Future research could focus on clinical trials to validate these findings and assess the real-world performance of these materials in vivo. Additionally, it would be useful to explore how other ions, such as magnesium (Mg^2+^) or zinc (Zn^2+^), contribute to the performance of bioactive materials, particularly in their role in remineralization and tissue repair.

## 5. Conclusions

The results of this study highlight the importance of environmental factors such as pH, temperature, and exposure time in determining the ion release profiles of bioactive restorative dental materials. The findings assist clinicians in selecting the most appropriate materials based on the specific needs of patients, especially those at risk of caries or dentine hypersensitivity. In conclusion, bioactive dental materials can contribute to the dental remineralization process and secondary caries prevention.

## Figures and Tables

**Table 1:** Type and chemical composition of the materials tested according to the available information from the manufacturer.

Material	Manufacturer	Type	Curing Mechanism	Composition	Bioactive Properties
**Cention Forte Filling** **Material**	Ivoclar (Schaan, Liechtenstein)	Alkasite	Self curing with light curing option	Ca-fluorosilicate glass, Ba-Al silicate glass, copolymer, Ca-Ba-Al fluorosilicate glass, 25–50% UDMA, 2.5–10% ytterbium trifluoride, 10–25% aromatic aliphatic UDMA, DCP and PEG-400-DMA	Releases hydroxide, calcium and fluoride ions
**Cention Primer**	Ivoclar (Schaan, Liechtenstein)	Self etching primer	Self curing	HEMA, MDP, Bis-GMA, D3MA, ethanol, methacrylate-modified polyacrylic acid, silicon dioxide, potassium hydroxide and campherquinone	
**Stela Self Cure**	SDI (Victoria, Australia)	Resin-based restorative material	Self curing	10–25% UDMA, 5–15% GDMA, 1–10% silica amorphous, 3–7% ytterbium fluoride, 1–5% MDP	Releases fluoride, calcium and strontium ions
**Fuji IX GP Fast**	GC Corp (Tokyo, Japan)	Glass ionomer	Self setting	Alumino-silicate glass, 25–<50% Polyacrylic acid, water, 5–<10% Tartaric acid	Stimulates remineralization through ion exchange
**Riva Light Cure HV**	SDI (Victoria, Australia)	High viscosity glass ionomer	Light curing	10–20% HEMA, 10–20% HEMA-phosphate derivative, 1–10% GDMA, 1–7% DMAEMA, 1–5% tartaric acid, 0–1% EDMAB, 0–1% camphorquinone, 0–1% BHT, >90% glass, oxide, 1–10% silica amorphous	Releases fluoride and strontium ions
**Riva Self Cure**	SDI (Victoria, Australia)	Glass ionomer	Self curing	20–30% acrylic acid homopolymer, 10–15% tartaric acid, 90–95% fluoro aluminosilicate glass	Releases fluoride and strontium ions
**Equia Forte HT Fil**	GC Europe (Leuven, Belgium)	Bulk fill glass hybrid	Self setting	95% fluoro aluminosilicate glass, 5% polyacrylic acid powder, reinforced with silicate particles, 25–<50% polyacrylic acid, 5–<10% polybasic carboxylic acid, 5–<10% tartaric acid	Promotes ion exchange; enhances remineralization and prevents demineralization

UDMA, uretanodimeethacrilate; Bis-GMA, bisphenol A-glycidyl methacrylate; MDP, 10-methacryloyloxydecyl dihydrogen phosphate; DCP, 1-(2,2-dimethylpropyl)–cyclopropene; PEG-400-DMA, Poly (ethylene glycol) dimethacrylate; HEMA, 2-hydroxyethyl methacrylate; GDMA, glycerol-dimethacrylate; DMAEMA, dimethylaminoethyl methacrylate; EDMAB, ethyl-4-(dimethylamino) benzoate T; BHT, butylated hydroxyanisol.

## Data Availability

The original contributions presented in the study are included in the article, further inquiries can be directed to the corresponding author.

## Supplementary Materials

The following supporting information can be downloaded at the following link: www.mdpi.com/article/10.3390/polym17050640/s1, Table S1: Average pH values with standard deviations (SD) observed for the different materials under three different acidity conditions, at two temperatures (44 and 37 °C) and at three different observation times (24 h, 7 days and 28 days).
