# Supplementary material for: The Effect of Different pH and Temperature Values on Ca2+, F−, PO43−, OH−, Si, and Sr2+ Release from Different Bioactive Restorative Dental Materials: An In Vitro Study
Source: Polymers (Basel). 2025 Feb 27;17(5):640. doi: 10.3390/polym17050640 (PMC11902544; doi:10.3390/polym17050640)
Supplement: Supplementary file 1 [file polymers-17-00640-s001.zip › polymers-3484787-supplementary.pdf]

Table S1: Average pH values with standard deviations (SD) observed for the different materials under three different acidity conditions, at two temperatures (44 and 37 °C) and at three different observation times (24 h, 7 days and 28 days).

Paramenters			Materials						
**pH**	**Time**	**T (°C)**	**Cention Forte Filling**	**Stela Self Cure**	**Riva Light Cure**	**Riva Self Cure**	**Equia Forte Ht Fil**	**Cention Primer**	**Gc Fuji Ix Gp Fast**
4.8	1 day	44	5.70±0.04	5.83±0.05	5.80±0.08	5.85±0.08	5.15±0.07	5.95±0.04	6.15±0.03
		37	5.02±0.06	5.22±0.03	5.12±0.04	5.22±0.07	4.87±0.03	5.28±0.07	5.42±0.07
	7 days	44	5.91±0.07	6.01±0.07	6.03±0.03	6.05±0.08	5.63±0.04	6.12±0.06	6.23±0.04
		37	5.75±0.03	5.85±0.05	5.85±0.06	5.90±0.06	5.42±0.07	5.95±0.05	6.07±0.06
	28 days	44	5.59±0.04	5.69±0.03	5.74±0.07	5.78±0.05	5.28±0.03	5.85±0.06	5.85±0.05
		37	5.53±0.04	5.65±0.05	5.64±0.05	5.65±0.03	5.18±0.05	5.74±0.07	5.86±0.03
6.8	1 day	44	5.61±0.08	5.71±0.06	5.70±0.07	5.75±0.07	5.84±0.04	5.79±0.03	5.90±0.07
		37	5.90±0.07	6.10±0.07	6.02±0.08	6.12±0.04	5.92±0.03	6.29±0.04	6.31±0.03
	7 days	44	6.31±0.07	6.41±0.04	6.41±0.05	6.45±0.05	6.29±0.04	6.47±0.06	6.64±0.06
		37	6.95±0.08	7.05±0.04	7.10±0.06	7.15±0.08	6.48±0.04	7.17±0.08	7.25±0.04
	28 days	44	6.53±0.03	6.63±0.03	6.66±0.07	6.73±0.05	6.13±0.07	6.68±0.06	6.97±0.03
		37	6.67±0.05	6.77±0.07	6.80±0.06	6.85±0.03	6.26±0.08	6.96±0.08	7.03±0.05
8.8	1 day	44	6.85±0.04	6.95±0.06	6.93±0.08	7.08±0.06	7.08±0.05	8.58±0.04	7.28±0.04
		37	7.36±0.04	7.50±0.03	7.5±0.03	7.55±0.08	7.28±0.08	7.63±0.07	7.73±0.04
	7 days	44	8.06±0.07	8.16±0.05	8.15±0.05	8.25±0.07	8.05±0.07	8.33±0.04	8.44±0.06
		37	8.02±0.05	8.12±0.05	8.11±0.04	8.22±0.04	7.59±0.08	8.25±0.03	8.30±0.07
	28 days	44	8.23±0.04	8.33±0.04	8.39±0.07	8.40±0.05	8.01±0.03	7.11±0.07	8.67±0.05
		37	7.68±0.06	7.78±0.07	7.86±0.03	7.94±0.06	7.64±0.05	7.95±0.06	8.09±0.03
